# Non-vitamin k antagonist oral anticoagulants in a European primary care physician survey

**DOI:** 10.3399/bjgpopen18X101602

**Published:** 2018-08-22

**Authors:** Claudio Cimminiello, Robert Hatala, Sami Pakarinen, Hernan Polo Friz, David Fitzmaurice, FD Richard Hobbs

**Affiliations:** 1 Professor, Studies and Research Center, Italian Society of Angiology and Vascular Pathology (Società Italiana di Angiologia e Patologia Vascolare), Milan, Italy; 2 Professor, Department of Cardiology, National Cardiovascular Institute and Slovak Medical University, Bratislava, Slovakia; 3 Professor, Department of Cardiology, Helsinki University Central Hospital, Meilahti Hospital, Helsinki, Finland; 4 Doctor, Department of Medicine, Vimercate Hospital, Vimercate, Italy; 5 Professor, Primary Care Clinical Sciences, University of Birmingham, Birmingham, UK; 6 Professor and Head, Primary Care Health Sciences, University of Oxford, Oxford, UK

**Keywords:** primary health care, atrial fibrillation, anticoagulants, surveys and questionnaires

## Abstract

**Background:**

Familiarity and competency in the options for stroke prevention in atrial fibrillation (AF) and the role of non-vitamin K antagonist oral anticoagulants (NOACs) may vary among primary care physicians (PCPs) from different European countries.

**Aims:**

To investigate PCP views on prescribing and managing NOACs across Europe and identify perceived unmet needs.

**Design & setting:**

Web-based survey including PCPs with particular interest in cardiovascular medicine.

**Method:**

A questionnaire was drawn up, containing 10 questions on initiation and ongoing management of NOACs; use of AF stroke guidelines on NOACs and anticoagulant switching; and perceived information needs.

**Results:**

The overall response rate was 42%. The majority of PCPs declared they are responsible for and confident in both initiating and managing NOAC therapy. In some countries, PCPs are not able to initiate NOAC therapy due to administrative barriers (namely, Italy and Slovakia). No single set of guidelines is referred to across all countries and over a fifth of responders indicate they do not follow specific guidelines. The main learning needs reported were more related to initiation than to ongoing management of anticoagulant therapy.

**Conclusion:**

According to this self-assessment survey, the experience of most PCPs in management of different aspects of AF appears good and only some felt the need for further training. However, in the light of the importance of this topic as public health issue, intensified efforts aiming at better equipping PCPs to meet their key roles in an integrated service across Europe are overdue.

## How this fits in

In addition to their central role in the diagnosis of AF, PCPs are responsible for much of the management of antithrombotic therapy needed for high stroke risk AF patients.

Familiarity and competency in the options for stroke prevention in AF and the role of NOACs may vary among PCPs from different European countries.

The results of this prospectively conducted web-based survey including PCPs with particular interest in cardiovascular medicine suggests that PCPs with a cardiovascular interest are prepared to assume more responsibility for managing anticoagulation.

Stroke prevention in AF is an important public health priority and better equipping PCPs to meet their key roles in an integrated service across Europe is warranted.

## Introduction

AF is the most common cardiac arrhythmia, but is often asymptomatic and unrecognised in primary care. However, screening for AF at the PCP level is able to detect a significant proportion of patients otherwise undiagnosed, the majority of whom are at high risk of stroke.^1^ An AF prevalence of approximately 3% in adults aged ≥20 years has been estimated, with greater figures in older persons.^2^ One in four middle-aged adults will develop AF in Europe and the US. AF is independently associated with a two-fold increased risk of all-cause mortality in women and a 1.5-fold increase in men, with direct costs of AF amounting to approximately 1% of total healthcare spending in the UK.^2^ These costs are deemed to increase dramatically unless AF is prevented and treated in a timely and effective manner.

In addition to their central role in the diagnosis of AF, PCPs are responsible for much of the management of antithrombotic therapy needed for high stroke risk AF patients. However, a lower percentage of AF patients at high stroke risk managed by PCPs receive anticoagulant therapy compared to those treated by specialists such as cardiologists.^3^ The advent of newer treatment options, the NOACs, also known as direct oral anticoagulants, has provided greater treatment choice for patients and clinicians. Several unexpected issues have emerged with NOAC use in primary care, such as inappropriate dosages of NOACs prescribed for a significant proportion of AF patients,^4^ and limited follow-up for NOAC patients since, unlike vitamin K antagonists (VKAs), NOACs do not need laboratory monitoring for dose adjustment.

Access restrictions to guideline-recommended therapies have been identified among other reasons for the suboptimal implementation of the guidelines.^5^ Since some European health systems place restrictions on the use of NOACs and PCPs are not authorised to prescribe them in some states (namely, Italy and Slovakia), it may be hypothesised that familiarity and competency in the options for stroke prevention in AF and the role of NOACs will vary among PCPs from different European countries. A survey was therefore conducted across several European countries to investigate PCP views and understanding on prescribing and managing NOACs, and to help identifying unmet learning needs.

## Method

A web-based survey was prospectively conducted on the behaviours and preferences of a group of PCPs with regard to AF stroke prevention with NOACs. A questionnaire consisting of 10 queries was set up. In February 2016, the questionnaire was sent by email to a total of 500 PCPs from France, Germany, Israel, Italy, Norway, Slovakia, Spain, Sweden, and the UK. The choice of these countries was made arbitrarily. The sample of doctors were selected by computerised randomisation from a database of PCPs with particular interest in cardiovascular medicine who, during previous scientific meetings and conferences, declared themselves available to participate in online surveys on the subject and whose professional characteristics had been recorded.

PCPs were eligible to take part if they were currently a practising PCP for not less than 3 years and not more than 30; spent more than 70% of their time in direct patient care (instead of activities such as administration, teaching, or research); and if they (and their family members) had never been affiliated with, or employed by, any pharmaceutical company as a consultant, employee, or researcher. The age and PCP setting (private or publicly-funded practice) were recorded as part of this survey but not used as criteria by which to exclude candidates from participation. Each responder received 20 Euros as compensation.

The questionnaire (Box 1) included 10 questions clustered in three main topics:

1. Responsibility and confidence in initiation and ongoing management of NOACs (4 questions).
2. Familiarity with AF stroke guidelines on NOACs use and anticoagulant switching (3 questions).
3. Perceived information needs, such as key topics and preferred education tools (3 questions).

Most questions were based on a multiple choice format and a list of the options can be seen in Box 1.

**Box 1.** Questions and potential responses of the survey

**(1) Responsibility and confidence in initiation and ongoing management of NOACs**	
Q1 Are you responsible for starting/initiating and/or managing anticoagulation therapy? Please select the one answer that applies:	a) Starting/initiating b) Managing c) Both
Q2 Do you feel confident in starting/initiating NOAC therapy in appropriate patients?	a) Yes b) No (with reasons for not being confident)
Q3 Do you feel confident in managing their ongoing care?	a) Yes b) No (with reasons for not being confident)
Q4 Who in your multidisciplinary team do you work closely with to manage anticoagulation therapy in your patients?	a) Cardiologist b) Neurologist c) Haematologist d) Internist d) Vascular physician e) Warfarin/anticoagulation clinic staff f) Nurse practitioner g) Pharmacist
**(2) Familiarity with AF stroke guidelines on NOACs use and anticoagulant switching**	
Q5 Which of the following guidelines do you apply to the clinical management of your patients with atrial fibrillation?	a) European Society of Cardiology guidelines 2016 b) European Heart Rhythm Association guidelines 2015 c) American Heart Association/American College of Cardiology/Heart Rhythm Society guidelines 2014 d) European Primary Care Cardiovascular Society guidelines 2015 e) Local/national atrial fibrillation management guidelines f) No specific guideline
Q6 What are the most common triggers for switching patients from a VKA (for example, warfarin) to a NOAC when clinically appropriate?	a) The burden of routine anticoagulation monitoring b) VKA adverse event c) Time in therapeutic range >70% d) Potential risk of VKA adverse event e) Patient preference f) Thrombotic event during VKA therapy g) Risk of discontinuation h) None of the above
Q7 From your experience, who is involved in switching patients from VKA therapy to a NOAC?	a) The PCP decides b) The PCP must refer to a specialist to switch c) Switching can happen at a thrombosis centre /anticoagulation clinic d) National resource allocator/market access decision maker is involved in the decision to switch e) None of the above
**(3) Perceived information needs**	
Q8 Are you interested in learning more about when or how to initiate anticoagulation therapy (NOACs or VKA), or its ongoing management?	a) Ongoing management of anticoagulation therapy b) When to initiate anticoagulation therapy c) How to initiate anticoagulation therapy d) None of the above
Q9 Which of the following topics do you feel is important to increase your knowledge, on a scale of 1-5 (where 1 = least important and 5 = very important)	a) The role of the primary care physician in the ongoing management of patients receiving NOACs b) The role of the primary care physician in prescribing NOACs c) The role of the anticoagulation clinic/thrombosis centre in the ongoing management of NOACs d) The role of the anticoagulation clinic/thrombosis centre in prescribing NOACs
Q10 Which of the following statements do you agree with when trying to find information about anticoagulation therapy?	a) Limited information on economic comparisons of the various anticoagulation therapies b) Lack of up-to-date real-world data c) Lack of adequate information from national scientific societies d) Lack of up-to-date clinical data e) Lack of adequate information from the pharmaceutical industry

AF = atrial fibrillation. NOAC = non-vitamin K oral anticoagulant. VKA = vitamin K antagonist.

The questionnaire was administered to participating PCPs over a 1-month period in February 2017. All questionnaire responses were anonymised before analysis. As no individual patient or practitioner health data were acquired in the survey, no ethical approval was necessary.

## Results

A total of 212 were returned out of 500 questionnaires sent out (42%), and all were available for study analysis. No differences were found between responders and non-responders in terms of the eligibility criteria. Characteristics of responders by country, years in practice, age range, and settings, are shown in Table 1.

**Table 1.** Distribution of responders by country, years in practice, age range, and settings

		All countries	France	Germany	Israel	Italy	Norway	Slovakia	Spain	Sweden	UK
Total		212	30	30	15	31	15	15	30	15	31
Years in practice	3–10	36	3	8	3	2	4	1	3	1	11
	11–20	76	9	11	6	11	5	6	12	4	12
	21–30	100	18	11	6	18	6	8	15	10	8
Age, years	<45	64	5	9	5	3	6	6	12	2	16
	45–54	77	14	13	5	16	6	5	9	2	7
	55–65	67	11	8	4	11	2	4	9	10	8
	>65	4	0	0	1	1	1	0	0	1	0
Setting	Community, general, or university hospital	80	4	1	5	7	3	3	26	9	22
	Private practice	132	26	29	10	24	12	12	4	6	9

### Initiation and ongoing management of NOACs

**Figure 1.**
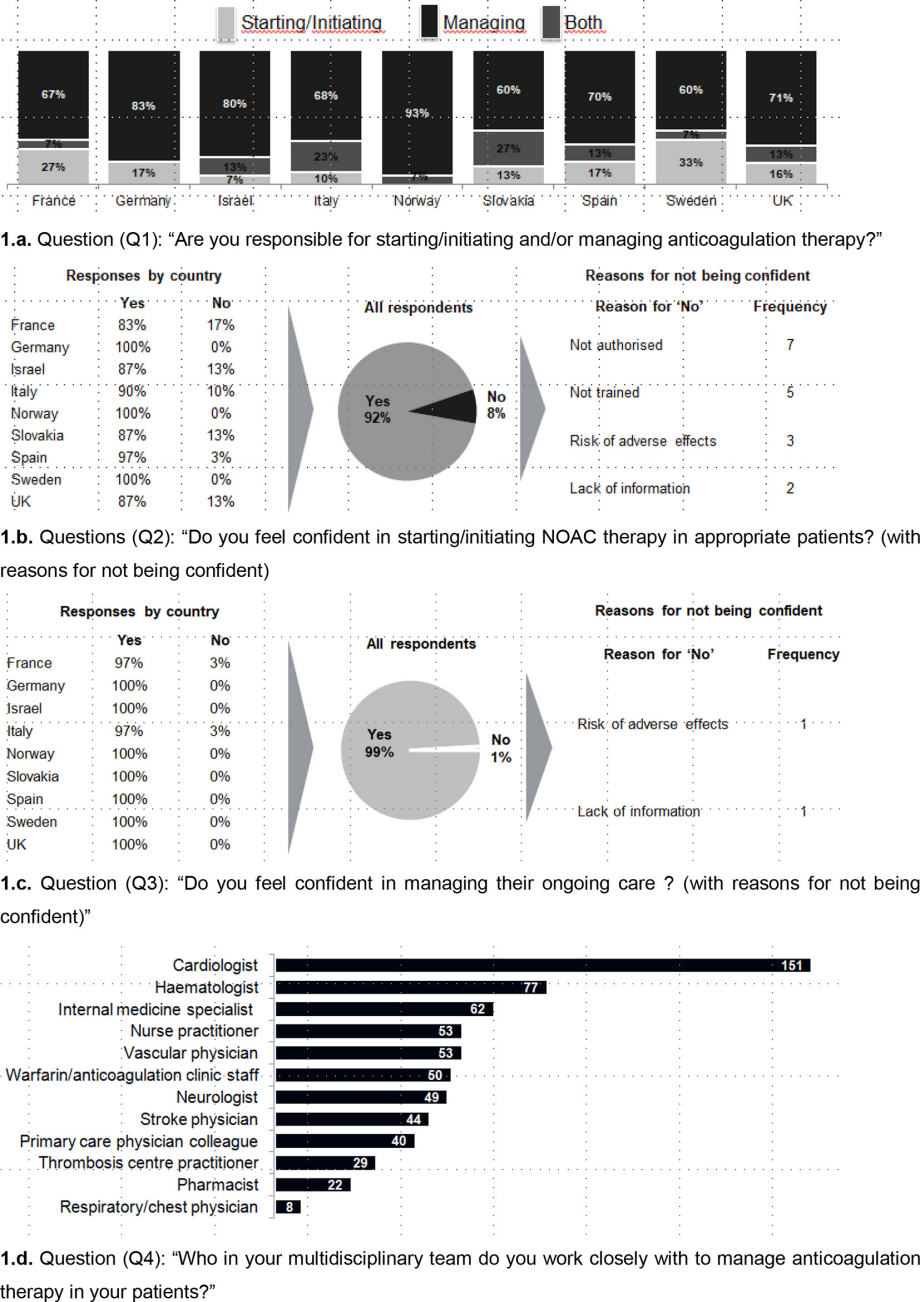
Responsibility and confidence in initiation and ongoing management of NOACs NOAC = non-vitamin K antagonist oral anticoagulant. VKA = vitamin K antagonist.

Most PCPs in each country declared they are responsible for both initiating and managing therapy (Figure 1a). France and Sweden have the largest proportion of PCPs who are only responsible for initiating (27% and 33% respectively). Italy and Slovakia have the largest proportion of PCPs who are only responsible for managing (23% and 27% respectively).

Ninety-two percent of responders declared they are confident in initiating NOAC therapy. All responding PCPs from Germany, Norway, and Sweden were confident, while in half of the countries (France, Italy, Israel, Slovakia, and the UK), >10% of responders stated they were not. Reasons for not being confident were 'not authorised' (*n* = 7), 'not trained' (*n *= 5), 'risk of adverse effects' (*n *= 3), and 'lack of information' (*n* = 2), as shown in Figure 1b.

PCPs reported that cardiologists are the most common management partners across all countries (Figure 1d).

### Guidelines and switching to a NOAC

**Figure 2.**
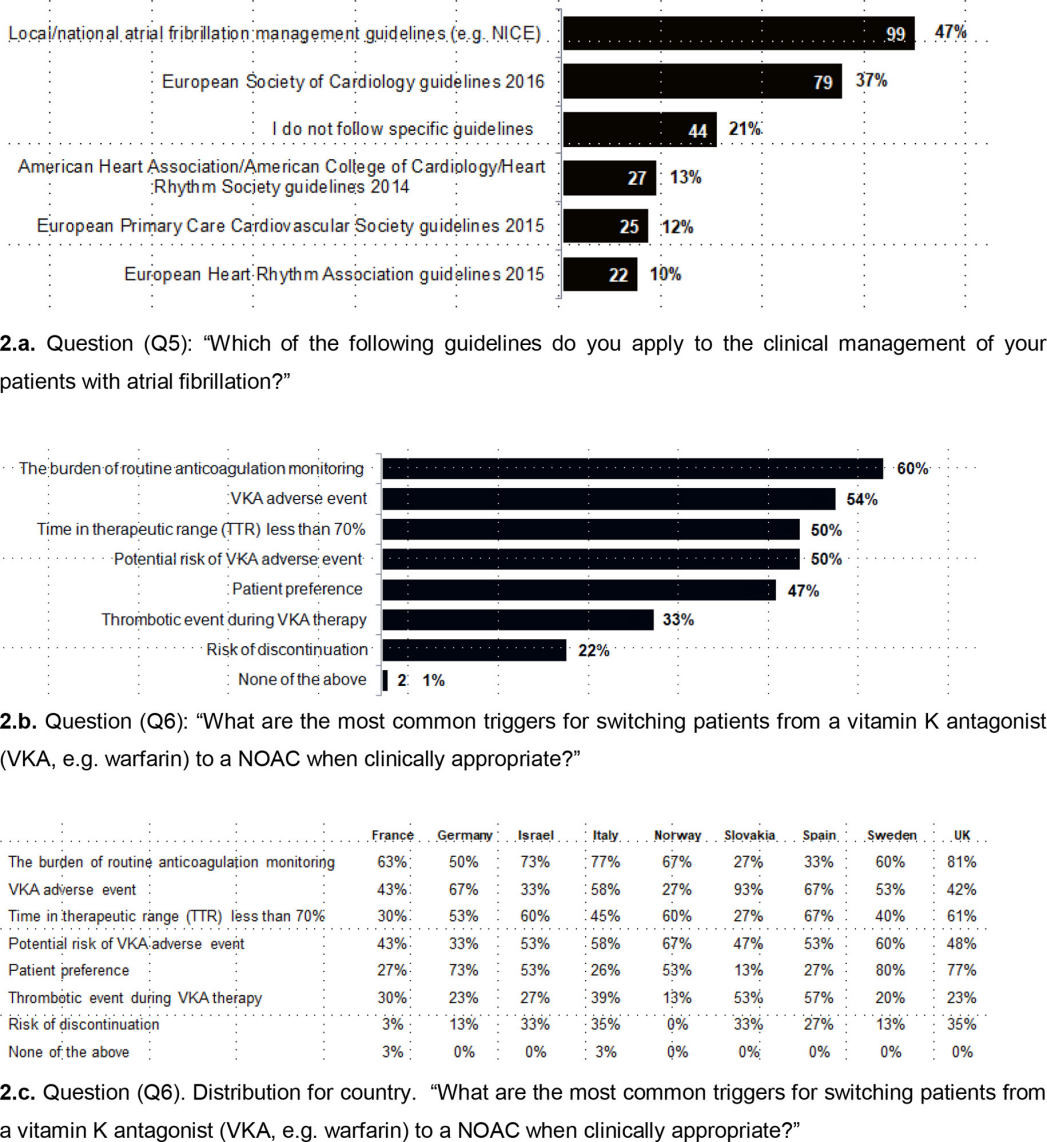
Familiarity with atrial fibrillation stroke guidelines on NOACs use and anticoagulant switching NOAC = non-VKA oral anticoagulant. VKA = vitamin K antagonist.

No single set of guidelines is referred to across all countries (Figure 2a). Local or national AF management guidelines are the most frequently cited choice but, importantly, just over one-fifth of responders indicate they do not follow specific guidelines.

In terms of reasons for switching from VKA to a NOAC, there is no a single driver (Figure 2b), although 60% of responders selected the burden of monitoring as a reason, with little to distinguish other reasons (including adverse events, potential adverse events, >70% of time in therapeutic range, or patient preference). Thrombotic events and risk of discontinuation are the least likely reasons for switching, but were still selected by one-third and one-fifth of responders respectively. The burden of routine monitoring is less of a driver in Slovakia and Spain than it is in other countries (Figure 2c). Adverse events are more likely to be a driver in Germany, Slovakia, and Spain than in other countries. Less than 70% of time in therapeutic range is less of a driver in France, Italy, Slovakia, and Sweden. Patient preference is a more frequent driver in Germany, Sweden, and the UK than in other countries.

**Figure 3.**
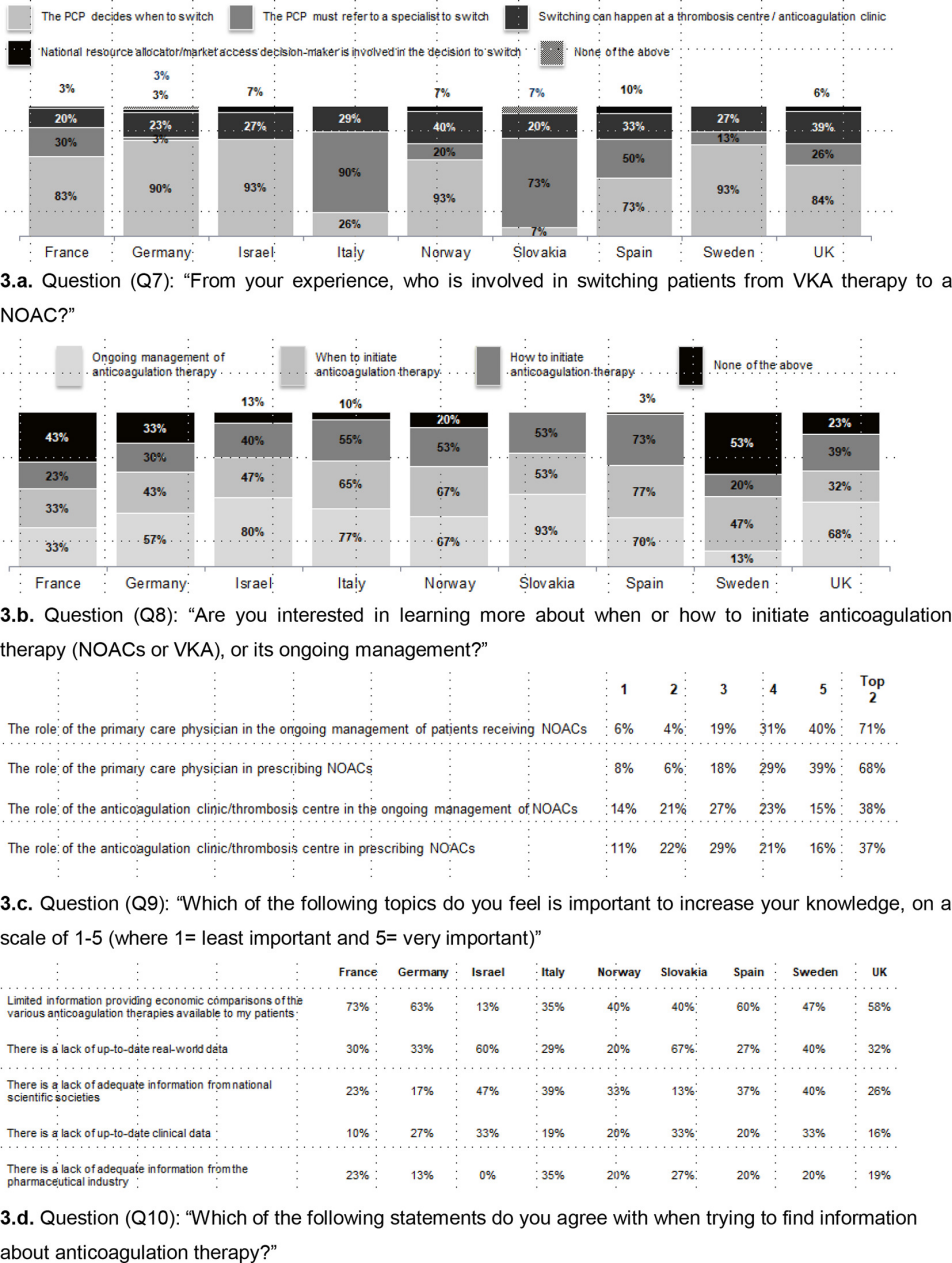
Perceived information needs NOAC = non-VKA oral anticoagulant. PCP = primary care physician. VKA = vitamin K antagonist.

Responders from most countries agreed that the PCP decides when to switch a patient from VKA to NOAC, with the exception of Italy and Slovakia (where PCPs are unable to prescribe NOACs) where PCPs refer to a specialist to switch. In Norway and the UK, it is slightly more common for switching to happen at a thrombosis centre or anticoagulation clinic than in other countries (Figure 3a).

### Information

The main learning needs reported were more on initiation than ongoing management of anticoagulant therapy (Figure 3b). In most countries, PCPs are interested in learning more about ongoing management, with the exception of France and Sweden. PCPs in Italy, Norway, and Spain are more interested in learning more about when to initiate, and those in Spain are more interested in how to initiate than in other countries. France and Sweden are more likely not to be interested in learning more on these topics. The responses on the importance for the PCPs of different educational topics are shown in Figure 3c.

Just over half of responders indicated there is an unmet need for economic comparisons of various anticoagulation therapies (Figure 3d). Approximately a third of responders agree that there is lack of up-to-date real-world data and lack of information from national scientific societies. Around a fifth of responders agree that there is a lack of up-to-date clinical data and lack of adequate information from the pharmaceutical industry. In terms of countries of origin, PCPs in France, Germany, Spain and the UK are more likely to agree that there is limited information providing economic comparisons of anticoagulation therapies. PCPs in Israel and Slovakia are more likely to agree that there is a lack of up to date real-world data. That there is lack of adequate information from national scientific societies is most strongly felt in Israel (Figure 3d).

## Discussion

### Summary

Better implementation of guidelines on antithrombotic therapy for AF in clinical practice is key to improving prognosis for all those at high risk of stroke.^6^ There are many barriers to such implementation and many of these apply to general practice, despite the role of primary care in AF stroke prevention being highlighted by international bodies.^5,7^ This requires a more systematic approach in health systems to the knowledge and access needs of PCPs. Furthermore, integration or shared care between of specialist and PCP roles is not well defined in guidelines or by health authorities, although proposals have been suggested.^8,9^ The scenario becomes even more complex in countries where administrative constraints are imposed on the use of NOACs, resulting in different roles for different clinicians.

This survey suggests that PCPs in Europe with an interest in cardiovascular disease feel confident in dealing with stroke prevention in AF and anticoagulation, with the exception of doctors of those countries (Italy and Slovakia) where prescription of NOACs by PCPs is prevented or restricted. The identification of the cardiologist as the preferential partner — as is also found in other surveys conducted between PCPs^10^ — should indicates the good level of knowledge on NOACs management by PCPs.

### Comparison with existing literature

There are, however, areas of controversy. Most PCPs state they do not follow a single guideline in this area, and 20% don’t follow guidelines at all. This under-use may be driven partly by what PCPs describe as discrepancies between guidelines for stroke prevention in AF, such as variations in practice patterns reported in a nationwide French survey.^11^ Furthermore, it is difficult to promote guidance to PCPs across Europe when no single set of guidelines exist.

The main drivers leading to switching from warfarin to NOACs were reported as the anticoagulation monitoring burden and perceived risks of bleeding, rather than non-optimal time in therapeutic range. These two main switching reasons were the same as those reported by PCPs in a Swiss survey.^12^ However, unlike the Swiss survey, the majority of responders here said they would manage the switch from warfarin to NOACs themselves, apart from PCPs in Italy and Slovakia and some PCPs in Spain, where most PCPs would refer switching to a specialist.

In terms of information needs, education on ongoing management of anticoagulant therapy was most popular (93% Slovakia, 80% Israel, 77% Italy), even though most had responded to being confident in this kind of ongoing management. In countries where the therapy initiation by PCPs is prevented, the PCPs unsurprisingly felt the need for initiation training. However, given that anticoagulation initiation is still markedly underachieved in general practice,^13,14^ training on this should probably be universal.

Importantly, the PCPs appear to accept that they should be assuming more responsibility for anticoagulation, rather than the traditional anticoagulation clinics, and the need for more training in this role was recognised. Finally, in terms of unmet needs, half of responders wanted more on economic comparisons between antithrombotic therapies, though some data exist.^15–17^

### Strengths and limitations

This study has some limitations. As with all questionnaires, perceptions of practice do not necessarily accord with actual practice. Since the authors tried to identify physicians actually involved in patient care and in the management of AF, it is not very surprising that they declare confidence in response to the survey’s clinical questions. Thus, this sampling is not necessarily representative of European practical doctors. Similar considerations apply to the limited representativeness of the number of PCPs responders and of arbitrarily chosen countries. Indeed, the heterogeneity of the answers contributes to highlight how the responders and their answers are complex. Questions on learning or unmet needs have not been explored using focus groups, semistructured interviews, or case stories. In addition, responders' views on switching back from NOACs to VKAs, or how and when to start NOACs, have not been addressed. Finally, the impact of other factors which may play a role when choosing an anticoagulant like pricing and reimbursement aspects — and even more so, the efficacy and safety as evidenced by clinical trials — were not asked about in this survey.

### Implications for practice

In their latest version, the European Society of Cardiology guidelines on AF propose an integrated approach to patient management by recognising the important strategic role of PCPs.^2^ According to these guidelines the specialist role is mainly supportive to that of PCPs. The perceived experience of most PCPs with cardiovascular interests in the management of different aspects of AF appears good^18^ and only a minority expressed the need for further training. Stroke prevention in AF is such an important public health topic that better equipping PCPs to meet their key roles in an integrated service across Europe is indicated, for example through further training. PCPs appear ready to take up the challenge.
